# Maternal sociodemographic characteristics of short and long interpregnancy intervals in Japan: an analysis using national birth data

**DOI:** 10.1186/s12889-025-24328-1

**Published:** 2025-09-24

**Authors:** Tasuku Okui

**Affiliations:** Medical Information Center, Kyushu University Hospital, 3-1-1 Maidashi, Higashi-ku, Fukuoka, 812-8582 Japan

**Keywords:** Infants, Interpregnancy intervals, Japan, National survey, Sociodemographic characteristics

## Abstract

**Background:**

Few studies have investigated the predictors of short and long interpregnancy intervals in Japan. Thus, this study investigated the maternal sociodemographic characteristics of short and long interpregnancy intervals among multiparous women using nationwide data in Japan.

**Methods:**

The data of the Longitudinal Survey of Newborns in the 21st Century (2010 Cohort) in Japan were used, and the interpregnancy intervals of mothers were calculated. The age group, marital status, number of births, employment status, educational attainment of mothers, and household income were used as explanatory variables. Interpregnancy intervals of less than 12 months and 60 months or more were defined as short and long interpregnancy intervals, respectively. A modified Poisson regression model was employed to investigate the relationships between short and long interpregnancy intervals and maternal characteristics, and unadjusted and adjusted analyses were conducted.

**Results:**

A total of 19,879 infants were used in the analysis. The results of the adjusted regression analysis showed that the risk ratio for short interpregnancy intervals increased with a decrease in maternal age, whereas the opposite trend was observed for long interpregnancy intervals. In addition, low educational attainment was positively associated with long interpregnancy intervals, and low household income was positively associated with short interpregnancy intervals. Employment status also played a role, with working mothers showing a negative association with short interpregnancy intervals and a positive association with long interpregnancy intervals.

**Conclusions:**

This study showed that maternal sociodemographic characteristics such as employment status, educational attainment, and household income were associated with short and long interpregnancy intervals in Japan.

**Supplementary Information:**

The online version contains supplementary material available at 10.1186/s12889-025-24328-1.

## Background

Interpregnancy intervals (birth spacing) refer to intervals between a live birth and the conception of the next pregnancy [1, 2]. Short interpregnancy intervals can cause problems, such as maternal nutritional depletion, folate depletion, and cervical insufficiency, and this can have adverse effects on the mother and fetus [3]. The World Health Organization (WHO) recommends an interpregnancy interval of at least 24 months [4]. Long interpregnancy intervals are associated with labor dystocia [3]. Short and long interpregnancy intervals have been associated with adverse birth outcomes, such as low birth weight, preterm birth, and small-for-gestational-age births [1, 5, 6]. In addition, short and long interpregnancy intervals are associated with child development and health, such as lower infant activity levels, developmental vulnerability in children, and higher diastolic blood pressure [7–9]. According to a nationwide epidemiological study in Japan [10], the associations have been shown between short and long interpregnancy intervals and preterm birth.

Short and long interpregnancy intervals are known to be affected by parental characteristics. Factors such as maternal employment status, educational attainment, contraceptive use, and pregnancy intentions have been shown to be associated with the interpregnancy intervals [9, 11–17]. Overall, low socioeconomic status tends to be associated with short interpregnancy intervals, although differences have been observed in the associations across countries [13]. In Japan, few studies have investigated the predictors of short and long interpregnancy intervals. A study investigated the characteristics of short interpregnancy intervals and discovered that the proportions of single-mother households and low educational attainments were higher in the group of short interpregnancy intervals [18]. However, the survey was conducted in a municipality, and the sample size was small. Interpregnancy intervals are related to factors related to family planning such as contraceptive use and fertility desire [19–21], and the relationship between interpregnancy intervals and sociodemographic factors can vary depending on countries. Therefore, it is important to investigate the predictors of short and long interpregnancy intervals using nationally representative survey data from Japan.

This study investigated the maternal sociodemographic characteristics of short and long interpregnancy intervals among multiparous women in Japan using nationwide data.

## Materials and methods

### Data

Data of the Longitudinal Survey of Newborns in the 21st Century (2010 Cohort) in Japan were used, and the data were provided from the Ministry of Health, Labour and Welfare following Article 33 of the Statistics Act in Japan. The longitudinal survey was conducted by the aforementioned Ministry in Japan; they investigated the longitudinal development of infants born in Japan from May 10, 2010 to May 24, 2010. We used the first and second survey data of the Longitudinal Survey of Newborns in the 21st Century (2010 Cohort) and the birth records (the Vital Statistics) of the surveyed infants. The first and second surveys were conducted in December 2010 and 2011, respectively. In the first survey, questionnaires were distributed to 43,767 respondents, and data were obtained on 38,554 infants (88.1%) [22]. In the second survey, questionnaires were distributed to 38,523 respondents, and data were obtained on 33,356 children (86.6%) [22].

We used information on the gestational age of the infant, number of fetuses, birth date of the infant and mother’s age, marital status, paternal nationality, and parity (number of births) in the birth record. In addition, we used information on the birth year and month of the siblings of the infants, employment status of mother, mother’s income, father’s income, and other incomes from the first survey. Maternal educational attainment was obtained from the second survey data. The maternal employment status covers that of one year prior to childbirth, and the income covers that in 2009 (previous year of the childbirth). Data on the second survey were used to obtain information on educational attainment, and data on infants who did not respond to the second survey were also used in the analysis. The educational attainment for mothers who did not respond to the second survey was treated as missing data.

### Explanatory variables

Maternal age group, marital status, number of births (parity), educational attainment, employment status, and household income were used as explanatory variables. These are representative sociodemographic characteristics which were shown to be associated with interpregnancy intervals in multiple previous studies in high-income countries [23–26]. The maternal employment status comprised unemployed, student, full-time worker, part-time worker, self-employed person, person doing piecework at home, and others. “Unemployed” and “student” were categorized as “unemployed,” and “self-employed person” and “person doing piecework at home” were categorized as self-employed persons. Household income was computed from the mother’s income, father’s income, and other incomes and categorized into quantiles. Maternal educational attainment comprised junior high school, specialized/professional training college (after graduation from junior high school), high school, specialized/professional training college (after graduation from high school), technical college/junior college, university, graduate school, and others. “Specialized/professional training college” and “high school” were categorized into “high school or training college,” and “university” and “graduate school” were categorized as “university or more.” Maternal age was categorized into under 25 years, 25–29 years, 30–34 years, 35–39 years, and 40 years or more. In addition, information on maternal marital status was derived from paternal nationality, which has a category of out-of-wedlock birth. The number of births and marital status were also used as explanatory variables. The number of births indicates that before the childbirth of this time and includes both live births and stillbirths.

### Outcome variables

The interpregnancy interval for each infant was calculated from the estimated conception date of the infant and the birth date of the closest sibling. The estimated conception dates were calculated from the birth dates and gestational ages of the infants. The definitions of short and long interpregnancy intervals vary across studies [9, 11, 13, 27, 28]. As previously mentioned, the WHO recommends an interval of at least 24 months [4]. However, 24 months is suggested to be unnecessarily long in high-income countries [1, 5]. We defined interpregnancy intervals of less than 12 months and 60 months or more as short and long interpregnancy intervals, respectively. This is because these benchmarks have been used as short and long interpregnancy intervals in previous studies [9, 13, 26] and have frequently been associated with adverse birth outcomes and pregnancy complications in high-income countries [1, 5, 29–31].

### Statistical analysis

Only women having past experience of live births were investigated because the interpregnancy intervals were surveyed. In addition, only data of singleton births were used in the analysis.

We tallied the number of births by the maternal characteristics and calculated the proportions of infants with short and long interpregnancy intervals. A modified (robust) Poisson regression model was employed to investigate the associations between short and long interpregnancy intervals and maternal characteristics [32]. Maternal age group, marital status, number of births, educational attainment, employment status, and household income were used as explanatory variables in the regression analysis. In addition to an adjusted analysis using the explanatory variables, an unadjusted analysis using each explanatory variable was conducted. Risk ratio (RR), robust variance, 95% confidence intervals (CI), and *p*-value were calculated, and *p*-value below 0.05 was judged to be statistically significant.

A complete-case analysis was conducted to deal with missing data, and multiple imputation was performed as a sensitivity analysis [33]. All the statistical analysis was conducted using R4.1.3 [34], and mice, lmtest, and sandwich were used as packages [33, 35, 36]. The statistics shown in this study were produced by the author using data that were provided from the Ministry of Health, Labour and Welfare, and those are not statistics published by the Ministry.

## Results

There were 38,554 infants who were enrolled in the study. After excluding 18,144 firstborn infants and 531 multiple births, data on 19,879 infants were used in the analysis.

Supplementary Table 1 indicates the number of study population and firstborn infants, which were excluded from the analysis, according to maternal characteristics. Similar to the study population, multiple births were also excluded for firstborn infants. Proportion of unemployed mothers was much higher in the study population compared with firstborn infants.

Table 1 shows the number and proportion of infants with short and long interpregnancy intervals according to maternal characteristics. The average interpregnancy interval was 26.0 months, and the median was 34.7 months in the data. The overall proportions of the short and long interpregnancy intervals were 9.6% and 13.8%, respectively. The proportion of short interpregnancy intervals increased with a decrease in maternal age, whereas the opposite was the case for long interpregnancy intervals. The proportion of long interpregnancy intervals was higher for non-married mothers than for married mothers, and it increased with an increase in the number of births. The proportions of short and long interpregnancy intervals were the highest in junior high school graduates in the educational attainments. The proportions of short and long interpregnancy intervals were the highest in “others” and “part-time workers,” respectively among employment statuses. The proportion of short interpregnancy intervals decreased with an increase in household income, whereas the reverse was the case for long interpregnancy intervals.

**Table 1:** Number and proportion of infants with short and long interpregnancy intervals by maternal characteristics

Maternal characteristics	All	Short interpregnancy interval		Long interpregnancy interval		Infants without information on interpregnancy interval
	Number	Number	Proportion (%)^b^	Number	Proportion (%)^b^	Number
Total	19,879	1,878	9.6	2,689	13.8	380
Maternal age group						
Under 25 years	1,101	322	30.0	11	1.0	29
25–29 years	4,687	719	15.6	264	5.7	79
30–34 years	8,083	552	6.9	892	11.2	128
35–39 years	5,190	257	5.0	1,207	23.7	99
40 years or more	818	28	3.6	315	40.8	45
Marital status						
Married	19,615	1,856	9.6	2,591	13.4	351
Non-married	264	22	9.4	98	41.7	29
Number of births						
One	13,990	1,364	9.9	1,541	11.2	229
Two	4,776	403	8.6	915	19.6	115
Three or more	1,113	111	10.3	233	21.6	36
Educational attainment						
Junior high school	736	91	13.1	154	22.2	41
High school or training college	8,645	846	10.0	1,368	16.1	157
Technical college or junior college	3,905	303	7.9	448	11.6	47
University or more	3,839	284	7.5	314	8.3	36
Others	46	4	8.7	4	8.7	0
Missing	2,708	350	13.4	401	15.4	99
Employment status						
Unemployed	10,480	1,261	12.2	848	8.2	142
Full-time worker	4,441	326	7.5	690	15.9	100
Part-time worker	3,728	155	4.3	958	26.4	99
Self-employed	955	68	7.2	161	17.1	16
Others	170	57	35.0	15	9.2	7
Missing	105	11	12.4	17	19.1	16
Household income^a^						
Quantile 1 (< = 383)	4,505	643	14.6	499	11.3	104
Quantile 2 (383–500)	4,709	483	10.4	545	11.8	82
Quantile 3 (500–672)	4,298	335	7.9	625	14.8	64
Quantile 4 (> 672)	4,493	209	4.7	698	15.8	64
Missing	1,874	208	11.5	322	17.8	66

aUnit: 10,000 yen

bProportion (%) was computed among infants with information on interpregnacy interval

Table 2 shows the results of the unadjusted regression analysis investigating the associations between short and long interpregnancy intervals and maternal characteristics. The RR for short interpregnancy intervals increased with a decrease in maternal age, while the reverse trend was observed for long interpregnancy intervals. Non-married status and number of births showed significantly positive associations with long interpregnancy intervals. In addition, lower educational attainment (junior high school and high school/training college) was significantly positively associated with short and long interpregnancy intervals. Regarding employment status, working mothers were negatively and positively associated with short and long interpregnancy intervals, respectively. Lower household income was positively and negatively associated with short and long interpregnancy intervals, respectively.

**Table 2:** The results of the unadjusted regression analysis investigating the associations between short and long interpregnancy intervals and maternal characteristics

Maternal characteristics	Short interpregnancy interval		Long interpregnancy interval	
	RR (95% CI)	*p*-value	RR (95% CI)	*p*-value
Maternal age group				
Under 25 years	4.56 (3.92, 5.31)	< 0.001	0.09 (0.04, 0.20)	< 0.001
25–29 years	2.39 (2.12, 2.70)	< 0.001	0.47 (0.40, 0.55)	< 0.001
30–34 years	Reference		Reference	
35–39 years	0.74 (0.63, 0.88)	< 0.001	2.20 (2.01, 2.41)	< 0.001
40 years or more	0.57 (0.38, 0.86)	0.008	3.58 (3.16, 4.05)	< 0.001
Marital status				
Married	Reference		Reference	
Non-married	1.10 (0.42, 2.86)	0.847	2.78 (1.83, 4.21)	< 0.001
Number of births	0.95 (0.87, 1.04)	0.257	1.40 (1.33, 1.46)	< 0.001
Educational attainment				
Junior high school	1.71 (1.33, 2.18)	< 0.001	2.66 (2.19, 3.22)	< 0.001
High school or training college	1.31 (1.15, 1.50)	< 0.001	1.93 (1.71, 2.18)	< 0.001
Technical college or junior college	1.05 (0.89, 1.23)	0.568	1.38 (1.20, 1.59)	< 0.001
University or more	Reference		Reference	
Others	1.34 (0.51, 3.51)	0.547	0.91 (0.30, 2.80)	0.873
Employment status				
Unemployed	Reference		Reference	
Full-time worker	0.61 (0.53, 0.70)	< 0.001	1.93 (1.73, 2.14)	< 0.001
Part-time worker	0.34 (0.28, 0.42)	< 0.001	3.45 (3.13, 3.80)	< 0.001
Self-employed	0.62 (0.47, 0.82)	< 0.001	2.12 (1.76, 2.55)	< 0.001
Others	2.89 (2.24, 3.72)	< 0.001	1.06 (0.60, 1.88)	0.844
Household income				
Quantile 1 (Lowest)	3.00 (2.54, 3.54)	< 0.001	0.73 (0.65, 0.82)	< 0.001
Quantile 2	2.27 (1.92, 2.69)	< 0.001	0.73 (0.65, 0.81)	< 0.001
Quantile 3	1.72 (1.44, 2.06)	< 0.001	0.94 (0.85, 1.05)	0.287
Quantile 4 (Highest)	Reference		Reference	

*RR* risk ratio, *CI* confidence interval

Table 3 shows the results of the adjusted regression analysis investigating the associations between short and long interpregnancy intervals and maternal characteristics. The RR for short interpregnancy intervals increased with a decrease in maternal age, whereas the opposite was the case for the RR for long interpregnancy intervals. Specifically, the adjusted RRs of under 25 years for short and long interpregnancy intervals were 4.23 (95% CI:3.58, 5.00) and 0.07 (95% CI:0.03, 0.15), respectively. The number of births was positively associated with short interpregnancy intervals, with an adjusted RR of 1.20 (95% CI:1.10, 1.30). In addition, junior high school graduates were negatively associated with short interpregnancy intervals, with an adjusted RR of 0.74 (95% CI: 0.57, 0.97), and lower educational attainment (junior high school, high school/training college, and technical college/junior college) was positively associated with long interpregnancy intervals, with adjusted RRs of 3.91 (95% CI: 3.23, 4.73), 2.09 (95% CI: 1.86, 2.36), and 1.44 (95% CI: 1.26, 1.65), respectively. Regarding employment status, working mothers were negatively and positively associated with short and long interpregnancy intervals, respectively. Specifically, adjusted RRs of full-time worker, part-time worker, and self-employed person for short interpregnancy intervals were 0.87 (95% CI: 0.75, 1.01), 0.33 (95% CI: 0.27, 0.41), and 0.71 (95% CI: 0.53, 0.94), respectively, and those for long interpregnancy intervals were 1.74 (95% CI: 1.56, 1.95), 3.13 (95% CI: 2.85, 3.44), and 1.72 (95% CI: 1.44, 2.05), respectively. Low household income was positively associated with short interpregnancy intervals, and adjusted RRs of the quantile 1, quantile 2, and quantile 3 were 1.90 (95% CI: 1.56, 2.32), 1.73 (95% CI: 1.43, 2.10), and 1.51 (95% CI: 1.25, 1.83), respectively. In contrast, a negative association was observed between the quantile 2 and long interpregnancy intervals, with an adjusted RR of 0.85 (95% CI: 0.76, 0.96).

**Table 3:** The results of the adjusted regression analysis investigating the associations between short and long interpregnancy intervals and maternal characteristics

Maternal characteristics	Short interpregnancy interval		Long interpregnancy interval	
	Adjusted RR (95% CI)	*p*-value	Adjusted RR (95% CI)	*p*-value
Maternal age group				
Under 25 years	4.23 (3.58, 5.00)	< 0.001	0.07 (0.03, 0.15)	< 0.001
25–29 years	2.27 (2.01, 2.57)	< 0.001	0.41 (0.35, 0.48)	< 0.001
30–34 years	Reference		Reference	
35–39 years	0.76 (0.65, 0.90)	0.001	2.14 (1.96, 2.33)	< 0.001
40 years or more	0.61 (0.40, 0.92)	0.019	3.23 (2.84, 3.67)	< 0.001
Marital status				
Married	Reference		Reference	
Non-married	1.20 (0.50, 2.85)	0.686	1.26 (0.83, 1.92)	0.282
Number of births	1.20 (1.10, 1.30)	< 0.001	1.03 (0.98, 1.09)	0.208
Educational attainment				
Junior high school	0.74 (0.57, 0.97)	0.027	3.91 (3.23, 4.73)	< 0.001
High school or training college	0.92 (0.80, 1.06)	0.246	2.09 (1.86, 2.36)	< 0.001
Technical college or junior college	0.94 (0.80, 1.10)	0.412	1.44 (1.26, 1.65)	< 0.001
University or more	Reference		Reference	
Others	1.08 (0.44, 2.62)	0.871	0.90 (0.29, 2.80)	0.858
Employment status				
Unemployed	Reference		Reference	
Full-time worker	0.87 (0.75, 1.01)	0.061	1.74 (1.56, 1.95)	< 0.001
Part-time worker	0.33 (0.27, 0.41)	< 0.001	3.13 (2.85, 3.44)	< 0.001
Self-employed	0.71 (0.53, 0.94)	0.016	1.72 (1.44, 2.05)	< 0.001
Others	3.08 (2.39, 3.98)	< 0.001	1.04 (0.60, 1.80)	0.902
Household income				
Quantile 1 (Lowest)	1.90 (1.56, 2.32)	< 0.001	0.91 (0.80, 1.02)	0.108
Quantile 2	1.73 (1.43, 2.10)	< 0.001	0.85 (0.76, 0.96)	0.006
Quantile 3	1.51 (1.25, 1.83)	< 0.001	0.99 (0.89, 1.09)	0.811
Quantile 4 (Highest)	Reference		Reference	

*RR* risk ratio, *CI* confidence interval

Supplementary Table 2 shows the results of the adjusted regression analysis investigating the associations between short and long interpregnancy intervals and maternal characteristics via multiple imputation. The results were similar to those of the complete-case analysis, whereas a significantly positive association was observed between full-time workers and short interpregnancy intervals and between the non-married status and long interpregnancy intervals in the multiple imputation analysis. In addition, significant associations were not observed between junior high school graduates and short interpregnancy intervals and between the quantile 2 of the household income and long interpregnancy intervals in contrast to the results of the complete-case analysis.

## Discussion

This study investigated the predictors of short and long interpregnancy intervals in Japan, and the associations of the intervals with certain factors were revealed. This section discusses the interpretations, implications, and limitations of the results.

Younger and older maternal age groups were positively associated with short and long interpregnancy intervals, respectively. Studies conducted in Australia, Canada, and African countries have observed similar associations [12, 13, 25, 29]. Fertility decline is pointed out to be associated with long interpregnancy intervals [13]. Fertility decreases at an advanced age [37], which can lead to the association between maternal age and interpregnancy intervals. It is also pointed out that the difference in contraceptive use depending on age was a reason for the associations [13]. In contrast, a study conducted in Denmark showed that older mothers were positively associated with short interpregnancy intervals [23]. In addition, another study conducted in the United States showed that older mothers were negatively associated with long interpregnancy intervals [26]. However, these previous studies in Denmark and the United States used maternal age at the preceding pregnancy, which can be a cause of the difference from the result of our study.

Furthermore, the number of births was positively associated with short interpregnancy intervals. Previous studies conducted in Ethiopia showed that a high number of births was negatively associated with short interpregnancy intervals compared with a low number of births [38, 39]. In contrast, studies conducted in Denmark and the United States showed that high parity was positively associated with short interpregnancy interval [23, 27], which aligns with the results of our study. In Japan, an experience of three or four pregnancies is a predictor of unplanned pregnancy [40], which was shown to be associated with short interpregnancy intervals in the United States [27, 41].

Household income was positively associated with short interpregnancy intervals, and previous studies conducted in countries including Australia and the United States have also revealed an association between income or socioeconomic level and short interpregnancy intervals [11, 24–26, 42]. A possible reason for the association is contraceptive methods, and the nonuse or poor knowledge of modern contraceptive methods has been associated with short or shorter interpregnancy intervals in both high-income and low- and-middle income countries [38, 39, 43, 44]. Contraceptive methods are affected by household income level, and high-income levels have been positively associated with the use of preferred or reliable contraceptive methods in studies conducted in Congo, Canada, and the United States [45–47]. In addition, unintended pregnancy was associated with low household income in a study conducted in Canada [48]. Regarding educational attainment, previous studies conducted in some sub-Saharan African countries discovered a positive association between high educational attainment and long interpregnancy intervals [13], pointing out that contraceptive methods are a possible reason for the association. In contrast, a study conducted in the United States showed a negative association between long interpregnancy intervals and high educational attainment [26], which aligns with the result of our study. In addition, a study conducted in Australia showed that higher educational attainment was associated with shorter interpregnancy intervals [25]. Therefore, it is likely that the relationships between educational attainment and long interpregnancy intervals differs between high-income and low- and-middle income countries. The reason for the positive association shown in this study should be investigated in a future study in Japan.

Working mothers were negatively and positively associated with short and long interpregnancy intervals, respectively, which aligns with the study conducted in Denmark [23]. Generally, women’s participation in the labor force is frequently associated with the postponement of childbirth and long interpregnancy intervals [13, 49–51], and postponement is often executed because of career planning and to earn more wages [52].

The results indicated that socioeconomic status was associated with short and long interpregnancy intervals in Japan. In particular, low income levels and educational attainment were associated with short and long interpregnancy intervals, respectively, and those are risk factors for adverse birth outcomes in Japan [53]. Thus, it is possible that low socioeconomic status and adverse birth outcomes are associated partially through interpregnancy intervals, and preventing short and long interpregnancy intervals may contribute to ameliorate the socioeconomic disparities in adverse birth outcomes in Japan. Therefore, the results in this study suggest that education on optimal interpregnancy intervals may be required particularly for low socioeconomic groups to prevent short and long interpregnancy intervals in Japan. In addition, a study investigating reasons for the association between lower educational attainment and long interpregnancy intervals in Japan is warranted in the future.

This study encountered some limitations. The information on maternal educational attainment was obtained through a survey performed 1.5 years after the birth of each infant. Thus, differences might exist in the actual educational attainment at the time of childbirth and that used in this study for some mothers. In addition, because this is a cross-sectional study, and the causal relationship between sociodemographic factors and interpregnancy intervals is not necessarily certain. Moreover, the survey whose data were used in this study targeted infants born in 2010, and the same survey has not been conducted by the government for recently born infants. It will be meaningful to investigate the association using more recent data in the future.

## Conclusions

We investigated the predictors of short and long interpregnancy intervals among multiparous women using nationwide data in Japan. Younger maternal age, high number of births, and low household income were associated with a higher risk of short interpregnancy intervals. In addition, older maternal age, low educational attainment, and being employed were associated with a higher risk of long interpregnancy intervals.

## Supplementary Information

Supplementary Material 1.

## Data Availability

The data that support the findings of this study are available from the Ministry of Health, Labour and Welfare in Japan but restrictions apply to the availability of these data, which were used under license for the current study and are not publicly available. Data are however available from the Ministry of Health, Labour and Welfare if the Ministry permits use of the data.

## Abbreviations

RR: Risk ratio
CI: Confidence intervals
WHO: World Health Organization
