# Supplementary material for: Maternal sociodemographic characteristics of short and long interpregnancy intervals in Japan: an analysis using national birth data
Source: BMC Public Health. 2025 Sep 24;25:3115. doi: 10.1186/s12889-025-24328-1 (PMC12461972; doi:10.1186/s12889-025-24328-1)
Supplement: Supplementary file 1 — Supplementary Material 1. [file 12889_2025_24328_MOESM1_ESM.pdf]

Supplementary Table 1. The number of study population and firstborn infants, which were excluded from the analysis, according to maternal characteristics.

Maternal characteristics	Study population Number (%)	Firstborn infants Number (%)
Total	19,879 (100.0)	17,952 (100.0)
Maternal age group		
Under 25 years	1,101 (5.5)	2,754 (15.3)
25–29 years	4,687 (23.6)	6,291 (35.0)
30–34 years	8,083 (40.7)	5,835 (32.5)
35–39 years	5,190 (26.1)	2,628 (14.6)
40 years or more	818 (4.1)	444 (2.5)
Marital status		
Married	19,615 (98.7)	17,575 (97.9)
Non-married	264 (1.3)	377 (2.1)
Number of births [a]		
One	13,990 (70.4)	58 (0.3)
Two	4,776 (24.0)	3 (0.0)
Three or more	1,113 (5.6)	0 (0.0)
Educational attainment		
Junior high school	736 (3.7)	460 (2.6)
High school or training college	8,645 (43.5)	6,925 (38.6)
Technical college or junior college	3,905 (19.6)	3,295 (18.4)
University or more	3,839 (19.3)	4,785 (26.7)
Others	46 (0.2)	46 (0.3)
Missing	2,708 (13.6)	2,441 (13.6)
Employment status		
Unemployed	10,480 (52.7)	3,704 (20.6)
Full-time worker	4,441 (22.3)	9,303 (51.8)
Part-time worker	3,728 (18.8)	4,197 (23.4)

Self-employed	955 (4.8)	544 (3.0)
Others	170 (0.9)	114 (0.6)
Missing	105 (0.5)	90 (0.5)
Household income [b]		
Quantile 1 (<=383)	4,505 (22.7)	3,178 (17.7)
Quantile 2 (383–500)	4,709 (23.7)	3,644 (20.3)
Quantile 3 (500–672)	4,298 (21.6)	3,993 (22.2)
Quantile 4 (>672)	4,493 (22.6)	5,618 (31.3)
Missing	1,874 (9.4)	1,519 (8.5)

a Number of births includes live births and stillbirths

b Unit: 10,000 yen

Supplementary Table 2. The results of the adjusted regression analysis investigating the associations between short and long interpregnancy intervals and maternal characteristics using multiple imputation.

Maternal characteristics	Short interpregnancy interval		Long interpregnancy interval	
	Adjusted RR (95%CI)	p-value	Adjusted RR (95%CI)	p-value
Maternal age group				
Under 25 years	3.97 (3.46, 4.55)	<0.001	0.08 (0.05, 0.15)	<0.001
25–29 years	2.13 (1.91, 2.38)	<0.001	0.46 (0.40, 0.52)	<0.001
30–34 years	Reference		Reference	
35–39 years	0.74 (0.64, 0.85)	<0.001	2.07 (1.91, 2.24)	<0.001
40 years or more	0.54 (0.38, 0.79)	0.001	3.24 (2.91, 3.62)	<0.001
Marital status				
Married	Reference		Reference	
Non-married	0.92 (0.62, 1.38)	0.693	1.86 (1.56, 2.21)	<0.001
Number of births	1.18 (1.10, 1.26)	<0.001	1.01 (0.97, 1.06)	0.549
Educational attainment				
Junior high school	0.87 (0.67, 1.14)	0.312	3.17 (2.58, 3.90)	<0.001
High school or training college	0.97 (0.84, 1.13)	0.73	2.01 (1.78, 2.27)	<0.001
Technical school or junior college	0.97 (0.81, 1.16)	0.744	1.43 (1.25, 1.64)	<0.001
University or more	Reference		Reference	
Others	0.94 (0.36, 2.51)	0.908	0.93 (0.37, 2.33)	0.872
Employment status				
Unemployed	Reference		Reference	
Full-time worker	0.86 (0.76, 0.98)	0.023	1.76 (1.59, 1.94)	<0.001
Part-time worker	0.35 (0.29, 0.41)	<0.001	2.91 (2.68, 3.16)	<0.001
Self-employed	0.70 (0.55, 0.88)	0.002	1.68 (1.44, 1.95)	<0.001
Others	2.97 (2.39, 3.70)	<0.001	1.10 (0.69, 1.75)	0.682
Household income				

Quantile 1 (Lowest)	1.76 (1.46, 2.13)	<0.001	0.96 (0.86, 1.08)	0.53
Quantile 2	1.58 (1.31, 1.89)	<0.001	0.92 (0.82, 1.02)	0.125
Quantile 3	1.41 (1.17, 1.70)	<0.001	1.00 (0.90, 1.11)	0.98
Quantile 4 (Highest)	Reference		Reference	

RR, risk ratio; CI, confidence interval
